# Expression of adiponectin, chemerin and visfatin in plasma and different tissues during a laying season in turkeys

**DOI:** 10.1186/s12958-015-0081-5

**Published:** 2015-07-31

**Authors:** Mélodie Diot, Maxime Reverchon, Christelle Rame, Pascal Froment, Jean-Pierre Brillard, Sylvain Brière, Gérard Levêque, Daniel Guillaume, Joëlle Dupont

**Affiliations:** INRA, UMR85, Unité Physiologie de la Reproduction et des Comportements, Nouzilly, F-37380 France; CNRS, UMR6175, Nouzilly, F-37380 France; Université François Rabelais, Tours, F-37041 France; IFCE, Nouzilly, F-37380 France; Fertilité et reproduction avicole (FERTIL’AVI), Rouziers-de-Touraine, F-37360 France; Hendrix Genetics-Grelier, Saint Laurent de la Plaine, F-49290 France

**Keywords:** Turkey, Adipokine, Laying season

## Abstract

**Background:**

In mammals, adipose tissue is able to secrete various hormones called adipokines including adiponectin (ADP), chemerin (Chem) and visfatin (Visf) which are involved in controlling energy metabolism as well as reproductive functions. Visf receptor is still unknown whereas ADP and Chem mainly act through AdipoR1, AdipoR2 and CMKLR1 and GPR1 receptors, respectively. No studies have yet demonstrated the presence of these three adipokines in peripheral tissues, ovarian cells or turkey plasma. Here, we investigated the expression (mRNA and protein) of ADP, Chem, Visf and their receptors in peripheral tissues and ovarian cells (granulosa and theca cells) from hierarchical follicles. Furthermore, we determined the plasma profile of ADP, Visf and Chem at different physiological stages: start, peak and end of the laying period in *Meleagris gallopavo* turkeys. This data was correlated with the metabolic data (plasma glucose, triglycerides, cholesterol and phospholipids).

**Methods:**

Tissue and ovarian cells mRNA and protein expression levels were determined by RT-qPCR and immunoblot, respectively. Plasma adipokines were measured by chicken ELISA and immunoblotting.

**Results:**

In turkeys, Chem is mainly expressed in the liver while ADP and Visf are mainly expressed in the abdominal adipose tissue and pectoral muscles,respectively. As in mammals, AdipoR1 and AdipoR2 expression levels (mRNA and protein) are highly present in muscle and liver, respectively, whereas the mRNA expression of CMKLR1 and GPR1 is ubiquitous. In ovarian cells, ADP, Visf, Chem and their receptors are more highly expressed in theca cells than in granulosa cells excepted for AdipoR1. Furthermore, we found that plasma levels of ADP, Chem and Visf were reduced at the end of the laying period compared to the start of this period. At the plasma levels, the levels of these adipokines are strongly negatively correlated with glucose and only plasma Chem is negatively correlated with cholesterol, triglycerides and phospholipids.

**Conclusions:**

In turkeys, ADP, Visf and Chem and their receptors are expressed in peripheral tissues and ovarian cells. Plasma concentration of ADP, Visf and Chem decrease at the end of laying period and only plasma Chem is negatively correlated with levels of cholesterol, triglycerides and phospholipids levels during the entire laying period.

## Background

In mammals, adiponectin (ADP), visfatin (Visf) and chemerin (Chem) are adipokines produced and secreted by white adipose tissue. They regulate glucose and energy metabolism [1] as well as reproduction including gonad steroidogenesis [2] and gonadotropin secretion [3, 4]. Adiponectin, one of the most studied adipokines, circulates in the form of homo-multimers [5] and its impact is mainly via binding to the AdipoR1 and AdipoR2 receptors [6]. Chem, or RARRES2, is a new adipokine that is involved in the regulation of adipogenesis, energy metabolism, and inflammation [7]. Chemokine-like receptor 1 (CMKLR1), also called ChemR23, GPR1 (G protein-coupled receptor 1) and chemokine (C-C motif) receptor-like 2 (CCRL2) are G-protein-coupled receptors known to bind Chem [8]. Visf is both a systemic adipokine and the cytosolic enzyme, nicotinamide phosphoribosyl transferase (Nampt) that is involved in metabolic (obesity, type II diabetes) and immune disorders [9]. In constrat to ADP and Chem, no Visf receptor has yet been identified.

Chicken exhibits particular features for glucose metabolism. For example, despite the presence of insulin circulating at “normal” concentrations, chickens present a high level of glycaemia, and low sensitivity to exogenous insulin [10–12]. In chickens, while ADP and its receptors and Visf have already been studied in plasma and different tissues [13, 14], Chem and its receptors have never been examined. In chicken plasma and tissues, ADP is predominantly a high molecular weight multimer [15]. Moreover, as in mammals, ADP is largely expressed in chicken adipose tissue [13, 16] and gonads [17]. ADP receptors, AdipoR1 and AdipoR2 are also expressed in different peripheral and reproductive tissues [18, 13]. As opposed to mammals, Visf is more highly expressed in muscle than in adipose tissue in chickens [19]. Consequently, Visf is described as a myokine and not an adipokine in chickens. In the latter species, the full-lentgh cDNA of Visf gene has been cloned and sequenced [20]. It shares a high amino acid sequence identity not only with the Visf of human but also fish. The chicken Visf gene expression is sex and tissue dependent. Indeed females have a higher amount of Visf mRNA in adipose tissue compared to males [21]. Moreover, Visf is expressed in gonads [14, 22] where it could play an important role in steroidogenesis, spermatogenesis and folliculogenesis [14]. Although ADP and Visf have been characterized in chickens, no data is available about the plasma concentration of these adipokines during the laying period in poultry.

In this study, we investigate the expression of these adipokines in peripheral tissues and ovarian cells and their plasma concentration in turkeys. Firstly, we studied the mRNA and protein expression of ADP, Chem, Visf and their receptors in peripheral tissues (adipose tissue, liver, pectoral and leg muscle and the heart) and ovarian cells (granulosa and theca cells) from hierarchical follicles. Secondly, we determined the plasma profile of ADP, Visf and Chem at different physiological stages: start, peak and end of laying period in *Meleagris gallopavo* turkeys (heavy strain Converter). Last but not least, this data was correlated with metabolic data (plasma glucose, triglycerides, cholesterol and phospholipids).

## Methods

### Animals

Eighty-eight *Meleagris gallopavo* turkeys (heavy strain Converter) from a commercial breeding unit of Hendrix Genetics (Saint Laurent de la Plaine, France) were studied at three different stages of a laying season: start (32 week-old), peak (35 week-old) and end (55 week-old). Animals were housed together with free access to feed and water and were exposed to a 15 h light:9 h darkness photoperiod. The diet nutritional composition was: Metabolisable Energy (2999 kcal/kg), crude protein (17 %), lysine (0.97 %), methionine (0.45 %), calcium (2.9 %), phosphorus (0.45 %). At the start, peak and end of the laying period, each fasted animal was weighted and a blood sample was taken. Blood samples were immediately centrifuged at 1,000 × g 15 min at 4 °C to obtain plasma that was then stored at -20 °C until use. The weight of animals was significantly decreased at the peak or at the end as compared to the start laying period (Table 1). At the end of laying period, six animals were sacrificed by electronarcosis and exsanguination and tissues (abdominal adipose tissue (AT), liver, heart, skeletal and pectoral muscles and ovarian hierarchical follicles) were collected and frozen at -70 °C until use. Granulosa (GC) and theca (TC) cells from F1 and F3/4 hierarchical follicles were dissected and also frozen at -70 °C. The experiment was carried out in accordance with French and European regulations on the care and welfare of animals in research. All procedures were approved by the Agricultural and Scientific Research Government Committee in accordance with the guidelines for the Care and Use of Agricultural Animals in Agricultural Research and Teaching (approval A37801).

**Table 1:** Body weight, plasma levels of glucose, cholesterol, triglycerides and phospholipids of turkeys (n = 88) at different periods of laying (start, peak and end)

	Start (n = 88)	Peak (n = 88)	End (n = 88)
Body Weight (kg)	12.462 ± 0.083^a^	12.176 ± 0.084^b^	12.009 ± 0.093^b^
Glucose (g/L)	1.991 ± 0.015^a^	2.249 ± 0.032^b^	2.541 ± 0.048^c^
Cholesterol (g/L)	1.702 ± 0.059^a^	1.801 ± 0.067^a^	1.890 ± 0.091^a^
Triglycerides (g/L)	15.086 ± 0.553^a^	16.383 ± 0.648^a^	11.380 ± 0.560^b^
Phospholipids (g/L)	5.235 ± 0.553^a^	4.919 ± 0.134^ab^	4.601 ± 0.132^b^

Different superscript letters indicate significant differences at *p* < 0.05

### Plasma biochemical parameters

The levels of plasma total cholesterol (g/L), triglycerides (g/L) and phospholipids (g/L) were measured by using the respective assays from Biolabo (Maizy, France). Plasma glucose levels were determined by using the GAGO-20 kit from Sigma Aldrich (Saint-Quentin Fallavier, France).

### Adipokines assays

Total plasma ADP, Visf and Chem levels were determined with commercially available chicken total ADP, Visf and Chem enzyme-linked immunosorbent assays (ELISA) (Chicken Total Adiponectin Elisa reference: E12A0125, Chicken Visf Elisa reference: E12V0003 and Chicken Chem Elisa reference: E12C0104), respectively, obtained from Hölzel Diagnostika (Koln, Germany). The measurements have been performed according to the manufacturer’s protocol, with an intra-assay coefficient of variation < 6 %. The sensitivity of the kits was 1 ng/mL for Visf, 0.1 ng/mL for total ADP and 1 pg/mL for Chem..

### RNA extraction and determination of ADP, Visf, Chem, AdipoR1, AdipoR2, CMKLR1, CCRL2 and GPR1 mRNA expression by RT-qPCR

Total RNA of different tissues (heart, liver, AT, pectoral and leg muscles) and GC and TC was extracted on ice with an ultraturax homogenizer in TRIzol® reagent according to manufacturer’s recommendation (Invitrogen™ by Life technologies™, Villebon sur Yvette, France). A treatment with DNaseI using the DNA-*free*™ Kit (Ambion® by Life technologies™, Villebon sur Yvette, France) was performed on the total RNAs. RNA quantity was assessed with a NanoDrop Spectrophotometer (Thermofisher, Villebon-sur-Yvette, France). Reverse transcription (RT) of total RNA (1 μg) was performed for 1 hour at 37 °C in a 20 μl mixture as previously described [23]. Real-time PCR using SYBR Green Supermix (Bio-Rad, Marnes la Coquette, France) and 250 nM of specific primers as described in Table 2 (Invitrogen™ by Life technologies™, Villebon sur Yvette, France) in total volume of 20 μl in a MyiQ Cycle device (Bio-Rad, Marnes-la-Coquette, France) was used to quantify adipokine and its receptor mRNA expression. Samples were tested in duplicate on the same plate and PCR amplification with water, instead of cDNA, was performed systematically as a negative control. After incubation for 2 minutes at 50 °C and a denaturation step of 10 minutes at 95 °C, samples were subjected to 40 cycles (30 seconds at 95 °C, 30 seconds at 60 °C, 30 seconds at 72 °C), following by the acquisition of the melting curve. Primers’ efficiency (E) was performed from serial dilutions of a pool of obtained cDNA and ranged from 1.80 to 2.00. Three reference genes were used: *RPL-15*, *ACTR3* and *EEF1A1*. For each gene, expression was calculated according to primer efficiency and Cq : expression = E^-Cq^. Then, relative expression of gene of interest/reference gene was analysed.

**Table 2:** Oligonucleotide primer sequences

Genes	Product size	Forward	Reverse
β-actin	188 bp	5’-ACGGAACCACAGTTTATCATC-3’	5’-GTCCCAGTCTTCAACTATACC-3’
RPL-15	194 bp	5’-TGTGATGCGTTTCCTCCTTGG-3’	5’-CCATAGGTTGCACCTTTTGGG-3’
EF1-α	90 bp	5’-AGCAGACTTTGTGACCTTGCC-3’	5’-TGACATGAGACAGACGGTTGC-3’
Visfatin	502 bp	5’-CGTTCAGCCCATTTGGTGA-3’	5’-AGTGGTGCCTCTGGACTTCG-3’
Chemerin	314 bp	5’-CGCGTGGTGAAGGATGTG-3’	5’-CGACTGCTCCCTAAAGAGGAACT-3’
CMKLR1	403 bp	5’-CGGTCAACGCCATTTGGT-3’	5’-GGGTAGGAAGATGTTGAAGAGGAA-3’
CCRL2	391 bp	5’-CACGCAGTGTTTGCTTTAAAAGC-3’	5’-CAACAGCCCACGTGACAATG-3’
GPR1	250 bp	5’-ACCTGCCTGAGGAAGAAGAA-3’	5’-AAAGGCCAGTGGAAGCCCAT--3’
Adiponectin	350 bp	5’-ACAGGTGCAGAAGGACCGAG-3’	5’-AAGACAGAGCCGCTTGCTTG-3’
AdipoR1	350 bp	5’-GAATACACACCGAGACGGGC-3’	5’-GCCCAAGACGCAGACAATGG-3’
AdipoR2	345 bp	5’-GAGACTGGCAACATCTGGAC-3’	5’-TGCGATGCCCAGGACACAAA-3’

#### Antibodies

**Fig. 1:**
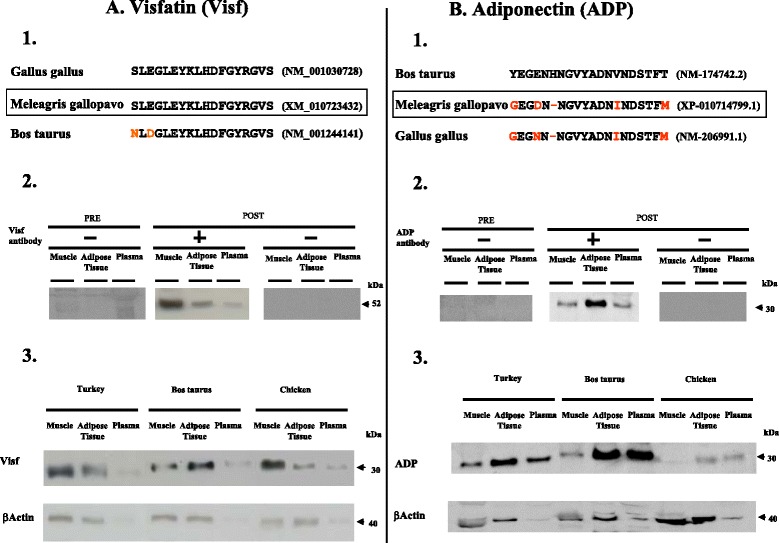
Charaterization of Visf (A) and ADP (B) antiserum. **1.** The sequence of the peptides used for immunisation is shown at the top followed by the corresponding Visf (A) or ADP (B) sequence in selected species. Red colour indicates difference from the peptide used for immunisation (Bos taurus peptide for adiponectin and Gallus gallus peptide for Visf). **2.** Plasma and protein lysates from chicken AT and leg muscle (60 μg) were analysed with SDS-PAGE under reducing conditions. Immunoblotting was performed with pre-immune rabbit serum (PRE) or chicken Visf antiserum (left panel) or Bos taurus ADP antiserum (right panel) (POST) in the absence (−) or presence (+) of 20 μg/ml of the chicken Visf antibody (left panel) or the Bos taurus ADP antibody (right panel). The arrow on the right indicates the position of the Visf (left panel) or ADP (right panel) signal. **3.** Visf (left panel) and ADP (right panel) protein expression by immunoblotting in leg muscle, AT and plasma in turkey, bovine and chicken species. The picture is representative of the lysates obtained on three different animals

The chicken and turkey anti-Visf antibody was produced by immunizing two rabbits with a 18-amino acid peptide corresponding to a region of chicken and turkey Visf located at the middle of the mature protein (Fig. 1a). When used in Western immunoblotting, the antiserum detected an approximately 52 kDa protein in turkey plasma, AT and leg muscle lysates resolved by reducing with SDS-PAGE (Fig. 1a). We demonstrated the specificity of the antiserum by showing that the 52 kDa signal in turkey plasma, AT and leg muscles lysates was eliminated when immunoblotting was performed in the presence of pre-immune rabbit serum or in the absence of chicken or turkey Visf antiserum (Fig. 1a). Furthermore, by using this Visf antiserum we confirmed that Visf is mainly expressed in muscles and not in adipose tissue in chicken. (Fig. 1a). For detection of ADP in turkey plasma, we used a bovine ADP antiserum that recognizes the ADP monomer (30 kDa) in both adipose tissue and plasma similarly to bovine and chicken species (Fig. 1b).

For detection of ADIPOR1 and ADIPOR2 we used antibodies from Abcam (Paris, France), Ab50675 and Ab40446, respectively. CMKLR1 (H160, SC66829) and CCRL2 (SAB2100371) antibodies were obtained from Sigma (Saint-Quentin-Fallavier, France) and Tebu-Bio (Le Perray-en-Yvelines, France), respectively.

### Detection and quantification of ADP, Visf, ADIPOR1, ADIPOR2, CMKLR1 and CCRL2 in turkey plasma and/or tissues by Western-Blot analysis

Lysates of tissues were prepared on ice with an ultraturax homogenizer in lysis buffer containing 10 mM Tris (pH 7.4), 150 mM NaCl, 1 mM EthyleneDiamineTetraacetic Acid , 1 mM Ethylene Glycol Tetraacetic Acid and 0.5 % Nonidet P-40 supplemented with various protease inhibitors (2 mM phenylmethanesulfonylfluoride, 10 μg/ml leupeptin, 10 μg/ml aprotinin) and phosphatase inhibitors (100 mM sodium fluoride, 10 mM sodium pyrophosphate, 2 mM sodium orthovanadate). Lysates were incubated on ice for 30 min before centrifugation at 12,000 × g for 20 min at 4 °C. The pellet was eliminated and the samples were stored at - 80 °C. The protein concentration of samples was measured using the bicinchoninic acid (BCA) protein assay (Interchim, Montluçon, France). Eighty micrograms of proteins were denaturated with Laemmli buffer for 5 minutes to 95 °C. Plasma samples (1 μL) were diluted with 10 μL of lysis buffer and mixed with sample buffer (final concentrations: 0.064 M Tris HCl, pH 6.8, 1 % SDS, 0.01 % bromophenol blue, 10 % glycerol). To convert the multimers of adiponectin to dimers and monomers, the samples were reduced at a final concentration of 200 mM dithiothreitol and denatured for 5 min at 95 °C. Denatured proteins extracts from tissue (80 μg) and plasma were submitted to electrophoresis in a 12 % (w:v) SDS-polyacrylamide gel. Non denatured plasma proteins were submitted to electrophoresis in a Tris-glycine gel 4-20 %. The fractionated proteins were transferred to a nitrocellulose membrane. The membranes were blocked with Tris-buffered saline containing 0.05 % Tween 20 (TBST) and 5 % milk for 30 min at RT. They were then incubated at 4 °C with appropriate primary antibodies at a 1/1000 final dilution. Finally, the blots were incubated for 1 h30 minutes at room temperature with a HorseRadish Peroxidase-conjugated anti-rabbit or anti-mouse IgG (dilution 1/5000). Proteins of interest were detected by enhanced chemiluminescence (Western Lightning Plus-ECL, Perkin Elmer, Villebon-sur-Yvette, France) with a G-box SynGene (Ozyme, St Quentin en Yvelines, France) and GeneSnap software (Ozyme, St Quentin en Yvelines, France). Then proteins were quantified by GeneTools software. The results were expressed as the intensity signal in arbitrary units after normalization.

### Statistical analysis

All experimental data are presented as means ± SEM. Two-way analysis of variance was used to test for differences in the expression levels of adipokines and their receptors in TC and GC at different follicle developmental stages. One-way analysis of variance was used to test for differences in plasma total adiponectin, Visf and Chem at different stages of laying period, in body weight, plasma levels of glucose, cholesterol, triglycerides and phospholipids of turkeys at different stages of laying period, and in peripheral tissue relative gene expression at the end of laying in turkeys. The level of statistical significance was set at *p* < 0.05. Statview software was used for all statistical tests. The relationships between quantitative parameters (plasma glucose, cholesterol, triglycerides and phospholipids concentrations) were investigated by Pearson’s correlation analyses, with the CORR procedure of SAS software.

## Results

### Expression of ADP, Chem, Visf, AdipoR1, AdipoR2, CMKLR1, CCRL2 and GPR1 in different peripheral tissues in turkeys

**Fig. 2:**
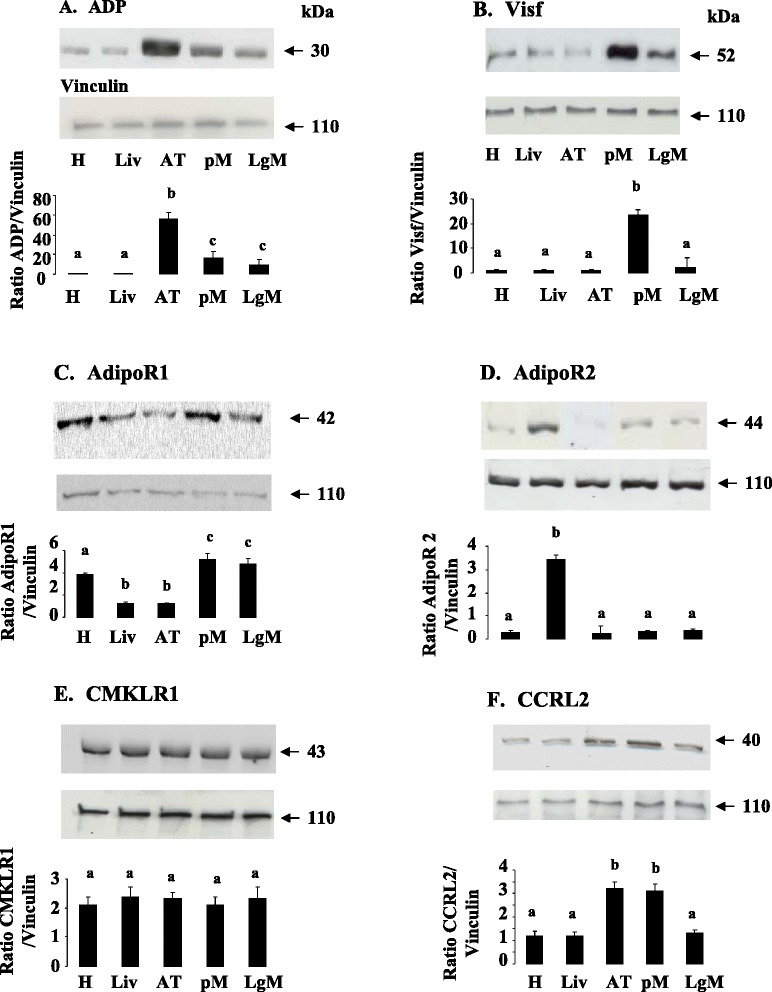
ADP (**a**), Visf (**b**), AdipoR1 (**c**), AdipoR2 (**d**), CMKLR1 (**e**) and CCRL2 (**f**) protein expression in peripheral tissues. ADP (**a**), Visf (**b**), AdipoR1 (**c**), AdipoR2 (**d**), CMKLR1 (**e**), and CCRL2 (**f**), protein levels were analyzed by western blotting in different peripheral tissues (Heart (H), Liver (Liv), Adipose Tissue (AT, pectoral (pM) and leg muscles (LgM) from six animals. Vinculin was used as a loading control. Data are represented as mean ± SEM (n = 6 animals). Different letters indicate a significant difference

We studied the expression of adipokines (ADP, Chem, Visf) and ADP and Chem receptors (AdipoR1, AdipoR2 and CMKLR1, CCRL2 and GPR1) by RT-qPCR in heart (H), liver (Liv), adipose tissue (AT), pectoral (pM) and leg (LgM) muscles in turkeys at the end of the laying period. As shown in Table 3, mRNA Visf was highly expressed in pectoral muscle as compared to adipose tissue, the liver, heart or leg muscles whereas mRNA Chem was mainly present in the liver and ADP in adipose tissue. As described in mammals, we observed that the expression of adiponectin receptors was tissue specific whereas those of CMKLR1 and GPR1 was ubiquitous. Indeed, AdipoR1 was highly expressed not only in pectoral muscle but also in leg muscle. At the opposite, AdipoR2 mRNA was mainly present in the liver. CCRL2 expression was higher in pectoral muscle and adipose tissue as compared to other tissues. We confirmed the results obtained by RT-qPCR at the protein levels through immunoblotting for ADP, Visf, AdipoR1, AdipoR2, CMKLR1 and CCRL2 (Fig. 2).We failed to detect Chem and GPR1 protein by immunoblot because we did not find good antibodies that recognize chicken Chem and GPR1.

**Table 3:** Peripheral tissue relative gene expression at the end of laying in turkeys^1^

	Heart	Liver	Adipose tissue	Pectoral muscle	Muscle of the leg
Visfatin	0.01	0.04	0.06	0.98	0.30
	±0.001	±0.012	±0.008	±0.277	±0.103
	a	a	a	b	a
Chemerin	0.23	3.69	0.35	0.17	0.31
	±0.090	±0.346	±0.172	±0.028	±0.090
	a	b	a	a	a
CMKLR1	0.003	0.001	0.002	0.003	0.003
	±0.001	±0.001	±0.001	±0.001	±1.10^−4^
	a	a	a	a	a
GPR1	0.02	0.01	0.02	0.03	0.02
	±0.002	±0.002	±0.003	±0.004	±0.004
	a	a	a		a
CCRL2	0.007	0.01	0.03	0.03	0.02
	±0.003	±0.005	±0.008	±0.004	±0.006
	a	a	b	b	a
Adiponectin	0.006	0.001	0.181	0.058	0.031
	±0.004	±0.001	±0.070	±0.024	±0.004
	a	a	b	a	a
AdipoR1	0.12	0.02	0.05	0.19	0.18
	±0.029	±0.003	±0.005	±0.037	±0.002
	a	b	b	c	c
AdipoR2	0.21	0.47	0.10	0.28	0.25
	±0.074	±0.068	±0.006	±0.020	±0.094
	a	b	a	a	ab

a-c means within a row without a common letter differ (*P* < 0.05).

^1^Relative expression was measured relatively to the geometric mean of 3 housekeeping gene expression (RPL-15, ACTR3 and EEF1A1) by real-time reverse-transcription PCR. Results are presented as means ± SEM. Genes and expression values written in bold indicate genes with difference in expression between tissues.

### Expression of ADP, Chem, Visf, AdipoR1, AdipoR2, CMKLR1, CCRL2 and GPR1 in ovarian cells in hierarchical follicles

**Fig. 3:**
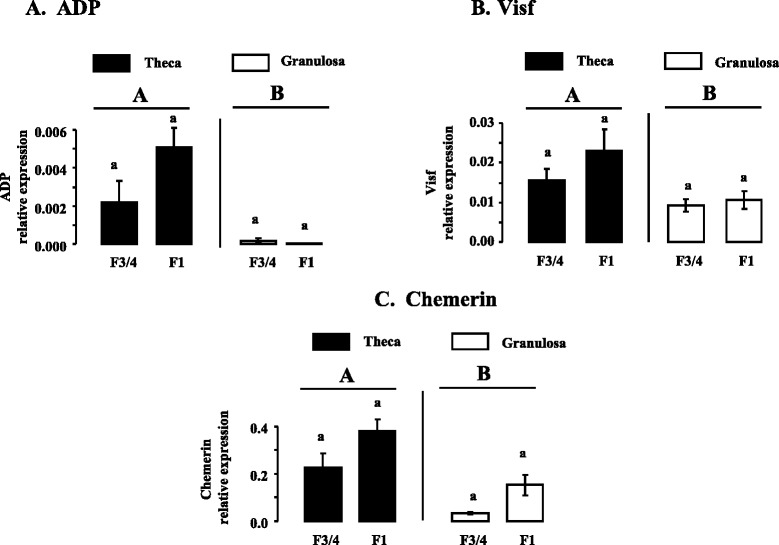
ADP (**a**), Visf (**b**), and Chem (**c**), mRNA expression in theca and granulosa cells from F3/4 and F1 hierarchical follicles. Relative expression of ADP (**a**), Visf (**b**), and Chem (**c**), mRNA in theca and granulosa cells from turkey F3/4 and F1 hierarchical follicles, based on real-time RT-PCR. Total RNA from all types of ovarian cells and hierarchical follicles was treated with DNAse and reverse transcribed as described in *Materials and Methods*. cDNAs were used in separate real-time quantitative PCR analyses of ADP, Visf and Chem mRNA or reference genes with SYBR green as the dye. ADP, Visf and Chem expressions were measured relative to the geometric mean of the expression of three reference genes as described in *Materials and Methods*. Results are means ± SEM from six different animals in each follicular stage. Different capital letters indicate a significant effect of the type of cells (Theca vs Granulosa cells) whereas lower case letters indicate a significant effect of the follicular stage (F3/4 vs F1)

**Fig. 4:**
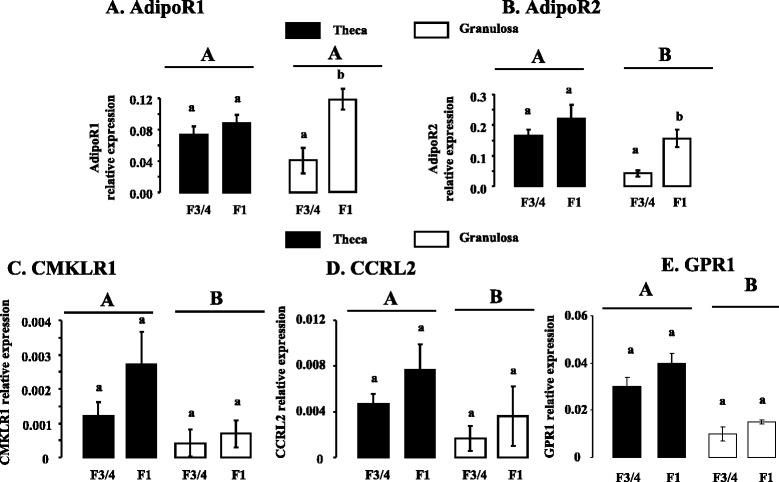
AdipoR1 (**a**), AdipoR2 (**b**), CMKLR1 (**c**), CCRL2 (**d**), and GPR1 (**e**), mRNA expression in theca and granulosa cells from F3/4 and F1 hierarchical follicles. Relative mRNA expression of adiponectin receptors (AdipoR1 and AdipoR2) and Chem receptors (CMKLR1, CCRL2 and GPR1) in theca and granulosa cells from turkey F3/4 and F1 hierarchical follicles, based on real-time RT-PCR. AdipoR1, AdipoR2, CMKLR1, CCRL2 and GPR1 mRNA expressions were measured relative to the geometric mean of the expression of three reference genes as described in *Materials and Methods*. Results are means ± SEM from six different animals in each follicular stage. Different capital letters indicate a significant effect of the type of cells (Theca vs Granulosa cells) whereas lower case letters indicate a significant effect of the follicular stage (F3/4 vs F1)

**Fig. 5:**
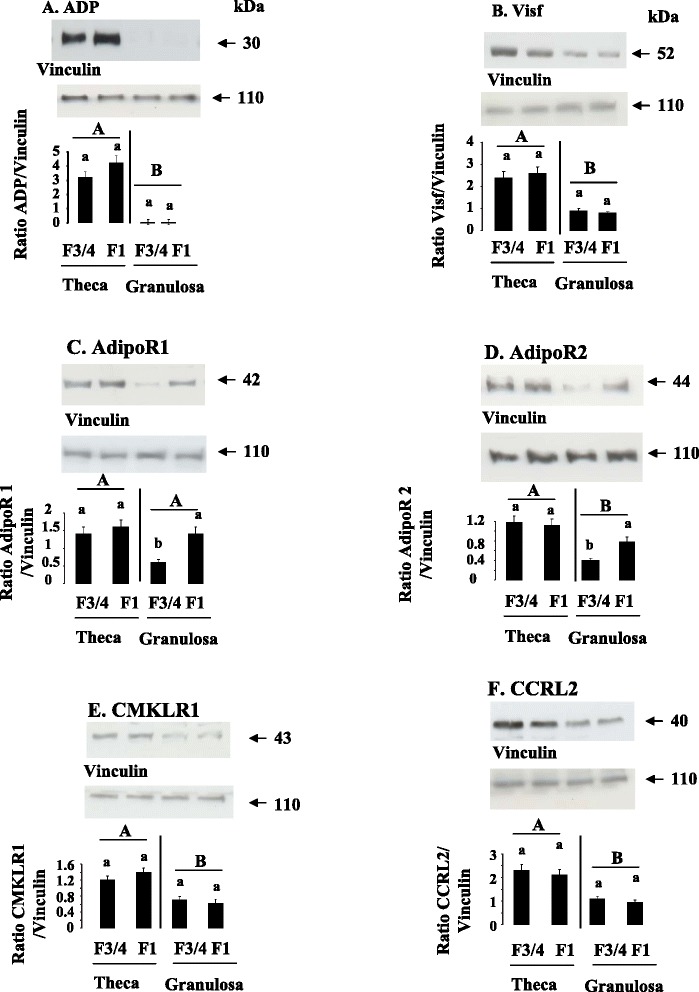
ADP (**a**), Visf (**b**), AdipoR1 (**c**), AdipoR2 (**d**), CMKLR1 (**e**), and CCRL2 (**f**), protein expression in ovarian cells. ADP (**a**), Visf (**b**), AdipoR1 (**c**), AdipoR2 (**d**), CMKLR1 (**e**), and CCRL2 (**f**), protein levels were analyzed by western blotting in theca and granulosa cells from F1 and F3/4 follicles. Vinculin was used as a loading control. Results are represented as means ± SEM (n = 6 animals). Different capital letters indicate a significant effect of the type of cells (Theca vs Granulosa cells) whereas lower case letters indicate a significant effect of the follicular stage (F3/4 vs F1). Data are represented as mean ± SEM. (n = 6 animals)

In mammals, some studies localized adipokines and their receptors in ovarian cells in different species [24–27]. In turkeys, we showed that mRNA expression of ADP, Chem and Visf was higher in theca cells compared to granulosa cells in both F1 and F3/4 hierarchical follicles (Fig. 3). Similar data was observed for AdipoR2, CMKLR1, CCRL2 and GPR1 receptors (Fig. 4). In both theca and granulosa cells, the expression of ADP, Chem and Visf was similar in F1 and F3/4 hierarchical follicles (Fig. 3). In contrast, in granulosa cells, AdipoR1 and AdipoR2 mRNA expression was higher in F1 than in F3/4 hierarchical follicles whereas CMKLR1, CCRL2 and GPR1 expression was unchanged irrespective of the differentiation stage of the follicle (Fig. 4). We confirmed the results obtained by RT-qPCR at the protein levels by immunoblotting (Fig. 5).

### Concentration of plasma ADP, Chem and Visf in turkey during a laying cycle

We next determined the plasma profile of adipokines (ADP, Chem and Visf) during a laying period in *Meleagris gallopavo* turkeys (heavy strain Converter) from a commercial breeding unit. We measured plasma levels of ADP and Visf at three different stages of laying (start, peak and end) by using a commercial Elisa chicken kit and by immunoblotting. For the methodology, we used a specific chicken and turkey anti-Visf antibody and a bovine anti-ADP antibody that cross-reacts with chicken and turkey species as described in the materials and methods. For the determination of plasma levels of Chem, we used only a commercial Elisa chicken kit since we found no antibodies suitable for immunoblotting.

**Fig. 6:**
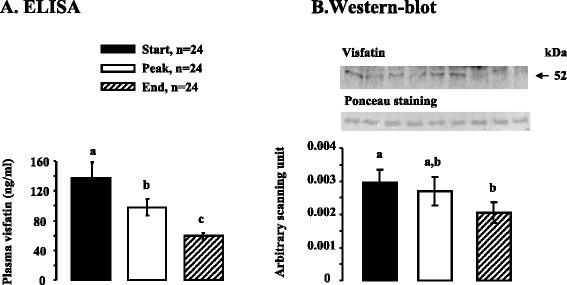
Plasma Visf concentration as determined by ELISA and immunoblot in turkey during a laying cycle. **a**. Plasma Visf levels in turkey at different stages of laying [start (S:32 week-old), peak (P: 35 week-old), end (E: 55 week-old)] based on ELISA. All serums samples used in Western blotting in denaturing conditions (**b**) contained equal amounts of protein; this was confirmed by staining the nitrocellulose membrane with Ponceau. Blots were quantified using an arbitrary scale. The letters above each bar indicate significant differences (*p* < 0.05). Data are represented as mean ± SEM. (n = 24/ stage of laying period)

**Fig. 7:**
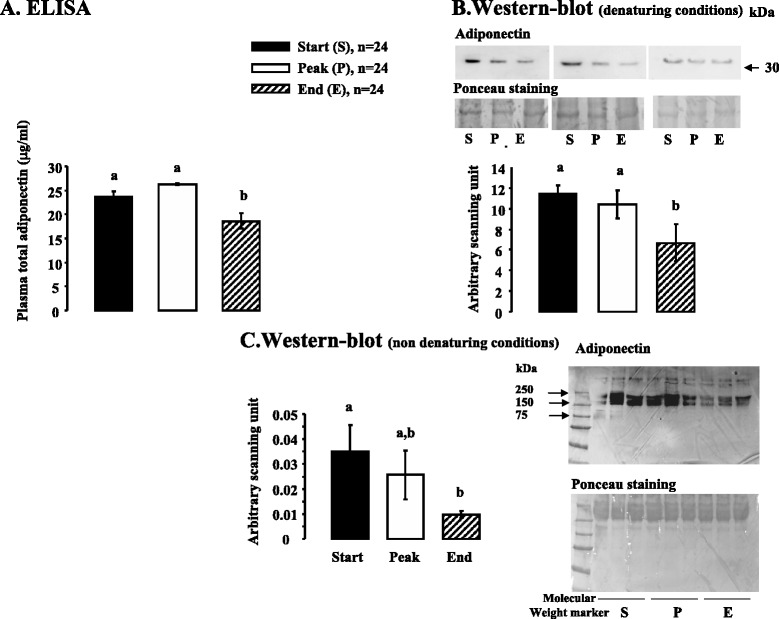
Plasma ADP concentration as determined by ELISA and immunoblot in turkey during a laying cycle. **a**. Plasma total ADP levels in turkey at different stages of laying [start (S:32 week-old), peak (P: 35 week-old), end (E: 55 week-old)] based on ELISA. All serums samples used in Western blotting in denaturing conditions (**b**) contained equal amounts of protein; this was confirmed by staining the nitrocellulose membrane with Ponceau. Blots were quantified using an arbitrary scale. The letters above each bar indicate significant differences (*p* < 0.05). Data are represented as mean ± SEM. (n = 24/ stage of laying period). **c.** Similar experiments were performed in **b.** in non denaturing conditions. The letters above each bar indicate significant differences (*p* < 0.05). Data are represented as mean ± SEM. (n = 24/ stage of laying period)

**Fig. 8:**
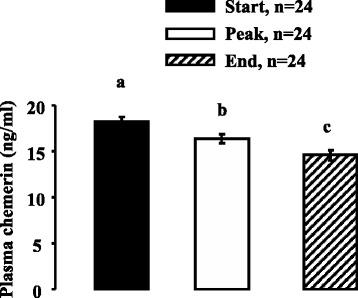
Plasma Chem concentration as determined by ELISA in turkey during a laying cycle. Plasma Chem levels in turkey at different stages of laying [start (S:32 week-old), peak (P: 35 week-old), end (E: 55 week-old)] based on ELISA. The letters above each bar indicate significant differences (*p* < 0.05). Data are represented as mean ± SEM. (n = 24/ stage of laying period)

As shown in Fig. 6, we observed by Elisa and immunoblotting that Visf plasma levels were significantly decreased at the end compared to the beginning of the laying period. Similar results were noted for the plasma levels of total ADP as determined using the Elisa kit (Fig. 7a) or for those of ADP monomeric (Fig. 7b) or hexameric forms (Fig. 7c) obtained by Western blot. Indeed, for example, in non denaturing conditions, we showed through immunoblotting that the hexameric form of ADP that is the main form revealed in these conditions is reduced by about three-fold at the end compared to the start of the laying period (Fig. 7c). As showed in Fig. 8, plasma Chem levels were also significantly reduced during the laying period.

### Correlations between adipokines concentrations and different blood parameters during a laying season

We next investigated whether the plasma concentration of ADP, Visf or Chem was correlated with plasma concentration of different blood biochemistry parameters such as glucose, cholesterol, triglycerides and phospholipids. Plasma glucose levels significantly increased during the entire laying period whereas plasma phospholipids and triglycerides levels decreased at the end (Table 1). On the contrary, plasma cholesterol levels remained unchanged (Table 1). To determine whether the plasma adipokines levels were correlated with those of plasma biochemistry parameters, a Pearson correlation coefficient test was performed. As showed in Table 4, we observed that during the entire laying season the plasma concentration of the three adipokines studied was negatively correlated with glycaemia. Moreover, plasma Chem levels were strongly negatively correlated with the plasma biochemistry parameters studied (glucose, cholesterol, triglycerides and phospholipids) whereas little significant differences were observed for ADP and Visf (Table 4).

**Table 4:** Coefficient of Pearson’s correlation

	Adiponectin	Chemerin	Visfatin
Glucose	−0.402	−0.5267	−0.223
	p = 0.01	p = 0.0004	p = 0.0534
Cholesterol	0.0197	−0.4812	0.0117
	p = 0.91	p = 0.0014	p = 0.93
Triglycerides	0.058	−0.4353	0.1469
	p = 0.73	p = 0.0045	p = 0.26
Phospholipids	−0.065	−0.39579	0.2137
	p = 0.707	p = 0.0104	p = 0.10

## Discussion

In this study, we showed for the first time that plasma adipokines including ADP, Visf and Chem decreased and were negatively correlated to plasma glucose during the laying period in turkey. Plasma Chem was also negatively correlated to plasma cholesterol, triglycerides and phospholipids. Furthermore, we observed that Chem was mainly expressed in the turkey liver while ADP and Visf were mainly expressed in the ATand pectoral muscles, respectively. In turkey, ADP, Visf and Chem and their receptors are expressed not only in peripheral tissues but also in ovarian cells. In ovarian cells, ADP, Visf, Chem and their receptors are more highly expressed in theca cells than in granulosa cells excepted for AdipoR1.

In this paper, for the first time, we studied the expression of Chem and its receptors, CMKLR1, GPR1 and CCRL2 in tissue and plasma in turkeys, a non-mammalian species. Chem also known as retinoic acid receptor responder protein 2 (RARRES2), is a 16 kDa protein secreted in an inactive form as proChem and is activated through cleavage of the C-terminus via inflammatory and coagulation serine proteases [28]. It is a novel adipokine that regulates adipocyte development and metabolic function [29]. In mammals, Chem expression is ubiquitous. However, several studies showed a higher expression of Chem in the liver as compared to other tissues in human and mice [30]. Furthermore, several reports have shown that liver injury may be associated with circulating Chem levels [31, 32] and that the liver may be a contributor to serum levels [33]. In turkeys, we observed that Chem is more highly expressed in liver than in AT or muscle. It will be interesting to study hepatic Chem expression during the fattening of birds, including ducks and hens. Active Chem exerts its action by binding to its extracellular receptor CMKLR1 on adipocytes and/or CCRL2 on activated macrophages or GPR1 on brain cells [34]. Only one study investigated the mRNA expression of Chem and its receptors in chicken adipose tissue [35]. Chem is expressed at higher levels in the abdominal fat of lean chickens compared to fat chickens [35]. Thus, Chem appears to be associated with leanness in chickens. On the contrary, a majority of human data indicate that plasma Chem is elevated in obesity/diabetes [36] and in inflammatory states [37, 38]. Animal studies reported the parallel findings in that obese and diabetic mice have elevated circulating levels of Chem [33, 39]. In turkeys, we showed that Chem plasma levels decrease during the laying period. Furthermore, they were negatively correlated with glycaemia and plasma cholesterol, triglycerides and phospholipids. As a limitation, it is acknowledged that the Elisa assay used in our study to measure plasma levels of Chem does not distinguish between active Chem and inactive proChem. Thus, total measured Chem concentrations might not equate to the actual amount of active Chem. In the plasma of healthy human, inactive proChem represents 80 % of total Chem [40]. This latter data is still unknown in birds. In the literature, the plasma and serum concentration of total Chem was reported to be about 3 to 4.4 nM in humans and 0.5 to 0.6 nM [41, 28] in mice [42]. Here, we determined a concentration of 1 nM in turkeys. In humans, plasma Chem levels are positively correlated with body fat, plasma glucose and triglyceride levels and lipid metabolism [43–46]. In our present study, we observed opposite results in turkey. Thus, as with chicken species, Chem could be associated with leanness and not with fattening in turkeys.

Similarly to Chem, we found that the plasma levels of Visf were also significantly decreased during the laying period. In birds, plasma Visf levels have been already determined by enzyme immunoassays in prepubertal and adult broiler chickens and hens [14, 22]. In chickens, plasma Visf levels are at least 28-fold higher in the adult chickens (about 300 ng/ml) compared with prepubertal male chickens (about 10 ng/ml) [14]. Furthermore, Krzysik-Walker et al., 2008 have observed that plasma Visf levels were significantly higher in 8-wk-old compared with 4-wk-old chickens [19]. On the contrary, we showed in our lab that in hens, Visf levels in plasma collected from adult animals (about 60 ng/ml at 52 week-old) were significantly lower than in plasma from young prepubertal (about 100 ng/ml at 3 weeks old) hens [22]. This latter data is consistent with our results obtained in turkeys showing a decrease in plasma Visf between the start (32 weeks old) and the end (55 weeks old) of the laying period. In chickens, Visf is considered to act more as a myokine than an adipokine [19]. We confirmed these results in turkeys as Visf is strongly expressed in muscles as compared to AT or the liver. However, we found that Visf is also expressed in ovarian cells and more precisely more in theca cells than in granulosa cells in turkeys. The role of Visf in these cells remains to be explored.

ADP is one of the most abundant plasma proteins in both birds and mammals. Unlike Visf, ADP exists in different molecular forms: monomer and multimers, which can be classified in high (18 or more ADP monomers), medium (a hexamer composed of two homotrimers) and low molecular weight (a homotrimer of three ADP monomers) [47]. In human and poultry, it is the high molecular weight form which is predominantly present and active in plasma [48, 15]. In turkeys, we showed by Elisa kit that total ADP plasma levels decrease at the end of laying period when animals lose weight and their glycaemia increase whereas their plasma triglycerides and phospholipids levels decreases. We confirmed these results by immunoblot in denaturing and non denaturing conditions. In these latter conditions, we observed bands of about 180 kDa suggesting that medium molecular weight (a hexamer composed of two homotrimers) isoforms are present in turkey plasma. In male chickens, plasma ADP levels were found to be significantly lower in 8-wk compared with 4-wk-olds and inversely related to abdominal fat pad mass [15]. In broiler chickens, Tahmoorespur et al (2010) showed that ADP mRNA expression in AT was inversely related to chicken belly fat deposition levels [49]. Furthermore, more recently, Yan et al., 2013 [50] observed that ADP inhibited lipid deposition and differentiation of chicken preadipocytes. An inverse relationship between plasma triglyceride and serum ADP has been described in humans [51]. In our study, we did not observe any significant correlation between plasma ADP and triglycerides in turkeys. However, we showed a negative correlation between glycaemia and plasma ADP that is already described in humans since ADP is known to increase insulin sensitivity. As in humans and mice, the chicken ADP gene is mainly expressed in adipose tissue [16]. However, it is also widely expressed in other tissues [16, 17]. We obtained similar data in turkeys. Indeed we found that ADP mRNA expression was the highest in AT, followed by the pectoral muscle and the heart and liver. As in humans and mice, we found that AdipoR1 is mainly expressed in muscle whereas AdipoR2 is more expressed in liver. We confirmed these results by Western blot in denaturing conditions.

Our data show that adipokines (ADP, Visf and Chem) and their receptors are present in ovarian cells in turkeys. More precisely, we observed that ADP expression is very low in granulosa cells. This data is consistent with those observed in humans [26, 27] or hen granulosa cells [18]. In this study, we found that AdipoR2 is higher expressed in theca cells as compared to granulosa cells. Furthermore, AdipoR2 is more expressed in F1 granulosa as compared to F3/4 granulosa suggesting that AdipoR2 could be involved in granulosa cell differentiation in turkeys. In human granulosa cells, our lab showed that AdipoR2 but not AdipoR1 could be implicated in the regulation of steroid production [52]. Thus, our results lead us to ask whether the adipokines could be fertility markers. In a recent work, we have showed that Visf decreases in vitro progesterone secretion by hen granulosa cells [22]. It will be interesting to correlate the plasma concentrations of adipokines with the fertility of animals (egg production but also embryo mortality). Expression of leptin receptor gene has been detected in the yolk sac of hen embryos [53]. It will be interesting to determine whether ADP, Visf and Chem and their receptors are present in the yolk sac and if their concentrations are correlated with parameters of fertility.

## Conclusions

In conclusion, we show for the first time that ADP, Chem and Visf are expressed not only in plasma and peripheral tissues but also in ovarian cells in turkeys. Furthermore, plasma levels of these adipokines decrease at the end of the laying period when the animals lose weight, their glycaemia increases and their triglycerides and phospholipids plasma levels decrease.
